# Emotional Eating and Weight Status in Adolescents: A Systematic Review

**DOI:** 10.3390/ijerph18030991

**Published:** 2021-01-23

**Authors:** Christine A. Limbers, Emma Summers

**Affiliations:** Department of Psychology and Neuroscience, Baylor University, One Bear Place #97334, Waco, TX 76798, USA; Emma_Greenwood@baylor.edu

**Keywords:** emotional eating, adolescents, review, weight status, BMI

## Abstract

Background: Despite evidence that emotional eating is associated with weight gain in adults, less is known about this association in adolescents. The purpose of the current study was to conduct a systematic review to assess the association between emotional eating and weight status in adolescents. This study also sought to describe existing measures of emotional eating in adolescents and explore weight-loss interventions that assessed emotional eating in relation to weight status in this population. Methods: Two independent reviewers searched the database PubMed for published or in press peer-reviewed studies that assessed the association between emotional eating and weight status in adolescents aged 12 to 19 years. Studies were excluded from this review if they were not written in the English language, did not include a measure of emotional eating, or were a dissertation study. Results: A total of 13 studies met full inclusion criteria and were included in the systematic review. Of the six longitudinal studies in the review, only one found a prospective association between emotional eating and weight status. The Dutch Eating Behavior Questionnaire was the most widely used measure of emotional eating in the systematic review (n = 6; 46.2%). The one intervention study included in this review found that baseline emotional eating was not associated with weight outcomes 2 years following gastric bypass surgery in obese Swedish adolescents (13–18 years). Conclusions: While there were some inconsistent findings across the studies included in this review, taken as a whole, the results largely do not support an association between emotional eating and elevated weight status or reduced weight loss in adolescents.

## 1. Introduction

Individuals who engage in emotional eating do so as a reaction to negative emotions rather than in response to feelings of hunger [1]. According to Psychosomatic Theory, emotional eating can serve as a temporary distraction from negative feelings [2,3]; however, because this distraction is only temporary, and does not facilitate directly confronting the source of one’s negative feelings, some individuals learn to engage in episodes of emotional eating over time as a way to cope with negative feelings. Since emotional eating typically involves the consumption of high-calorie foods, there has been increasing interest in whether this maladaptive coping strategy increases one’s risk for overweight/obesity [4]. Frayn and Knauper (2018) conducted a systematic review to evaluate the associations between emotional eating and weight outcomes in adults. Across the longitudinal and intervention studies included in their review, the authors found that emotional eating was associated with weight gain and reduced weight loss in adults over time [5].

To the best of our knowledge, there has not been a systematic review to date that has assessed the association between emotional eating and weight status in adolescents. Adolescence is an important period to investigate the relationship between emotional eating and weight status since it has been identified as a critical period for the development of emotional eating [6,7], and eating patterns that form during adolescence often persist into adulthood and affect long-term health outcomes [8]. In addition, given that emotional eating during adolescence has been identified as a potential precursor to more severe disordered eating [9], it is critical to understand other possible adverse outcomes associated with emotional eating which may further underscore the importance of prevention and early intervention. Given the absence of a systematic review in this area, the purpose of the current study was to conduct a systematic review to assess the association between emotional eating and weight status in adolescents. In order to inform future research and clinical work related to emotional eating in adolescents, this study also sought to describe existing self-report measures of emotional eating in adolescents and explore weight-loss interventions that assessed emotional eating in relation to weight status in this population.

## 2. Methods

The methodology for this systematic review was guided by the recommendations of Petticrew and Gilbody [10]. Using the search terms “emotional eating,” “adolescent,” “weight,” “BMI,” “weight loss intervention,” “longitudinal,” and, “teen,” the electronic database PubMed was searched in October 2020. Published or in press peer-reviewed studies were included in this systematic review if they assessed the association between emotional eating and weight status in adolescents aged 12 to 19 years. If a study included any participants outside this age range, it was excluded from the review. Studies were also excluded from this review if they were not written in the English language, did not include a self-report measure of emotional eating, or were a dissertation study. Two independent reviewers performed the search using article titles and abstracts. Reference lists of articles and dissertation studies were searched for additional studies that met inclusion criteria. Discrepancies between reviewers were resolved through discussion.

**Figure 1:**
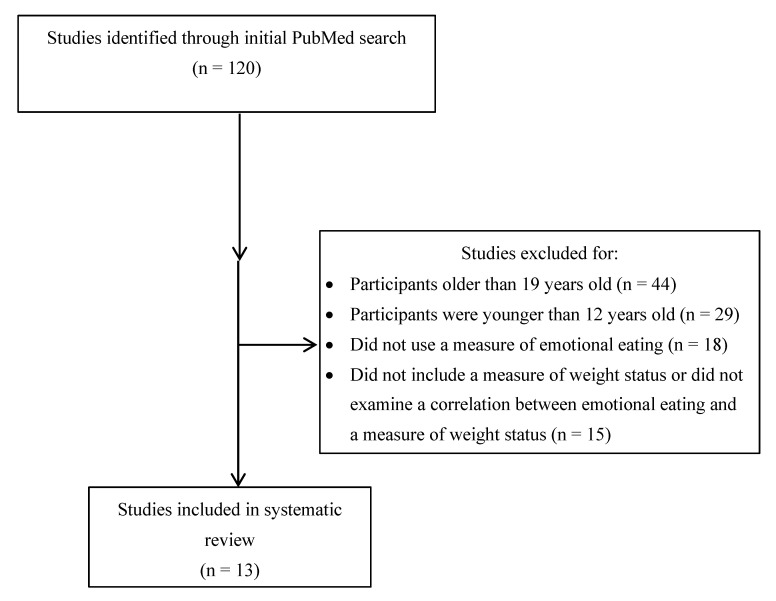
Flow Diagram of Article Selection.

Studies included in this review were organized into a summary table (Table 1). The table was broken down into the following categories: Emotional Eating Measure, Sample Demographics (when available sample size, percentage female, mean age, age range, percentage race/ethnicity, mean BMI, percentage in normal, overweight, obese BMI groups), Country of Study, Longitudinal Study, Weight Status Finding, and Intervention Study. As indicated in Figure 1, a total of 120 articles initially resulted from the search. After thoroughly reading each article, 44 studies were excluded because participants were older than 19 years old, 29 studies were excluded because participants were younger than 12 years old, 18 studies were excluded because they did not use a measure of emotional eating, 15 studies were excluded because they did not include a measure of weight status or did not examine a correlation between emotional eating and a measure of weight status, and 1 study was excluded because the full-text article was not available. As a result, a total of 13 studies met full inclusion criteria and were included in the present systematic review.

## 3. Results

### 3.1. Study Descriptives

The majority of the studies included in this review (n = 10; 76.9%) were conducted in the year 2013 or later. The largest number of studies were carried out in the U.S. (n = 6; 46.2%). Other countries where included studies were conducted were Canada (n = 1; 7.7%), Italy (n = 1; 7.7%), Netherlands (n = 2; 15.4%), Portugal (n = 1; 7.7%), China (n = 1; 7.7%), and Sweden (n = 1; 7.7%). The sample size ranged from 72 to 1197 across the included studies. Six studies (46.2%) included a longitudinal study design and six were cross sectional (46.2%); 1 study (7.7%; [11]) assessed adolescents pre-gastric bypass surgery and at a 2 year follow up. The majority of the studies included a community (n = 10; 76.9%) or college sample (n = 2; 15.4%) of non-treatment-seeking adolescents. The gastric bypass study by Järvholm and colleagues was the only treatment-seeking sample of adolescents included in our review. All of the studies included both male and female adolescents except 1 (7.7%) [12], which was comprised of female college students. Of the studies that reported race/ethnicity data (n = 7), five included a predominantly White or European sample and two examined emotional eating in predominantly Latino adolescent samples in the U.S. [7,13].

### 3.2. Associations between Emotional Eating and Weight Status

Of the six longitudinal studies included in this review, one [14] found a prospective association between emotional eating and weight status. In this study, the authors found that emotional eating at age 16 years was positively associated with percent body fat at age 19 years among 153 adolescents from the U.S. The other five longitudinal studies did not find a prospective association between emotional eating and weight status. It should be noted that in one of the longitudinal studies included in our review [15], despite not finding a prospective association between emotional eating and weight status, the authors reported that baseline emotional eating was positively associated with baseline weight, body mass index, waist circumference, and DXA fat mass index.

In the six cross-sectional studies included in this review, one [16] demonstrated some support for an association between emotional eating and weight status in adolescents. Laghi and colleagues (2015) reported a significant association between emotional eating and BMI in Italian adolescent males in their sample; this same association was not reported for adolescent females. The other five cross-sectional studies included in this review did not find an association between emotional eating and weight status.

### 3.3. Measures of Emotional Eating

The Dutch Eating Behavior Questionnaire was the most widely used measure of emotional eating in the studies included in the systematic review (n = 6; 46.2%). Other measures of emotional eating utilized were the Three Factor-Eating Questionnaire (n = 3; 23.1%), the Emotional Eating Scale (n = 3; 23.1%), and the Motivation for Eating Scale (n = 1; 7.7%).

### 3.4. Interventions and Emotional Eating

As noted above, one intervention study was identified in our review that assessed the association between emotional eating and weight status. Järvholm and colleagues (2018) assessed the association between emotional eating and weight status in obese Swedish adolescents (13–18 years) undergoing gastric bypass surgery. They found that baseline emotional eating was not associated with weight outcomes 2 years following gastric bypass surgery. Although higher levels of emotional eating two years post gastric bypass surgery were significantly correlated with decreased weight loss at the 2 year follow up, this correlation was in the small range.

## 4. Discussion

The purpose of this study was to conduct a systematic review to assess the association between emotional eating and weight status in adolescents. This study also sought to describe existing measures of emotional eating in adolescents and explore weight-loss interventions that assessed emotional eating in relation to weight status outcomes in this population. While there were some inconsistent findings across the studies included in the review, taken as a whole, the results largely do not support an association between emotional eating and elevated weight status or reduced weight loss in adolescents. Only one of the six longitudinal studies [14] reported a prospective association between emotional eating and changes in percent body fat. In this study, the follow-up period (3 to 4 years) was longer than the follow-up period in the majority of the other longitudinal studies included in this review. This finding, in conjunction with recent findings from the systematic review which reported an association between emotional eating and weight gain and reduced weight loss in adults over time [5], suggests that it may take a longer period of time to begin to see the impact of emotional eating on weight status in an individual. That is, there may be a cumulative effect of emotional eating over time that places an individual at greater risk for developing obesity. While firm conclusions cannot be drawn from the findings of one study, our systematic review highlights the need for more longitudinal research in adolescents evaluating the prospective association between emotional eating and weight status over longer follow-up periods. Longitudinal studies that assess the association between emotional eating and weight status across adolescence and young adulthood are also needed.

Most studies included in our review were conducted in the year 2013 or later, which reflects that this is an emerging area of research. Some of the inconsistent findings across the studies in our review likely can be attributed to methodological differences. For example, the studies included in the review were carried out across several different countries and used different measures of emotional eating. The nature of the samples also varied and included community samples of adolescents, college students, and treatment-seeking obese adolescents. Some studies included in our review did not provide race/ethnicity information about the samples; however, those studies that did were predominantly conducted with White or European adolescents. Given the disproportionate rates of overweight/obesity among racial minority adolescents in the U.S., our findings underscore a need for more research on emotional eating and weight status with racially and ethnically diverse adolescents. In one study included in our review [13], the authors found that acculturative stress was associated with higher levels of emotional eating in a sample of Latino adolescents from the U.S. Thus, it may be important for future research that investigates emotional eating and weight status in racial minority adolescents to consider assessing other constructs such as acculturative stress and perceived discrimination. Another consideration for future research in this area is the translation of emotional eating measures for adolescents into languages which may facilitate the assessment of emotional eating with more diverse adolescent populations.

While the majority of the studies included in the systematic review did not find a direct association between emotional eating and weight status in adolescents, the study by Stojek and colleagues (2017) highlights that other eating behaviors when interacting with emotional eating may increase the risk for adolescent weight gain. In this study, the authors found that the interaction between baseline emotional eating and baseline loss of control eating (i.e., the perception that one cannot control what and how much they eat) predicted increased adiposity and disordered eating 1 year later. Future research is needed to elucidate the interactive effect between emotional eating and other eating behaviors such as loss of control eating that may predict increased adiposity among adolescents.

Four different measures of emotional eating were utilized across the studies in our review, with the Dutch Eating Behavior Questionnaire used most widely. The development and validation of emotional eating measures specifically designed for use in children and adolescents are a relatively new area of research [17]. One limitation of existing measures of emotional eating developed for use in adolescents, which may ultimately serve as a barrier to the assessment of this construct in this population, is the length of these measures. For example, the Dutch Eating Behavior Questionnaire is 33 items long and the Emotional Eating Scale for Children and Adolescents is 25 items long. To address this issue, a short form of the Emotional Eating Scale for Children and Adolescents was recently developed [18]. This short form has demonstrated good internal consistency reliability, a high degree of overlapping variance with the original Emotional Eating Scale for Children and Adolescents Total Score and Subscale Scores, construct validity, and a unidimensional factor structure [18,19].The existence of a brief, psychometrically sound measure of emotional eating for adolescents may facilitate the assessment of this construct in weight-loss interventions as well as population-wide studies that focus on adolescent health.

Järvholm and colleagues (2018) was the only intervention study included in our review that assessed emotional eating in relation weight status outcomes. The authors found that baseline emotional eating was not associated with weight outcomes 2 years following gastric bypass surgery in obese adolescents from Sweden. Researchers have called for the development of interventions that specifically address emotional eating in the context of weight-loss interventions [20]. While these interventions have been largely used with adult populations, we identified two interventions through conducting this search that did not meet inclusion criteria for our systematic review but are worth noting because of their potential to address emotional eating in adolescents. Boutelle and colleagues (2018) tested the initial efficacy of the Preventing Emotional Eating Routines (PEER) Program, which was a program that integrated emotion regulation skills with parenting techniques and behavioral weight loss principles, in adolescents who were overweight or obese. The authors found that the intervention resulted in significant decreases in emotional eating and trends towards significance for weight loss and reductions in emotion dysregulation [21]. Kamody and colleagues (2019) assessed the implementation of a 10 week dialectical behavioral therapy group intervention to address subthreshold levels of maladaptive eating behaviors in adolescents. The authors found that the group led to decreases in adolescent emotional eating and binge eating [22]. Taken together, findings from these two studies are promising and suggest interventions that specifically target maladaptive eating behaviors in adolescent populations have the potential to lead to reductions in emotional eating.

While the majority of studies in our review included a mixed sample of male and female adolescents, one study included in the review [16] suggests that it may be valuable to assess the association between emotional eating and weight status separately in male and female adolescents. Laghi and colleagues (2015) reported a significant association between emotional eating and BMI in Italian adolescent males in their sample, but this same association was not reported for adolescent females. This finding is consistent with previous research that has found some differences with regard to how male and female adolescents interpret some items on the Emotional Eating Scale for Children and Adolescents [23]. Potential reasons for gender differences in emotional eating between males and females include differences in rates of depression and the utilization of different coping styles. Future research in this area should take into account potential gender differences and examine whether gender impacts the association between emotional eating and weight status in adolescents.

There were a number of limitations to the present systematic review. While the review was carried out by two independent reviewers using strict inclusion/exclusion criteria, conducting a meta-analysis may have provided a more rigorous methodology for synthesizing the findings of the studies reviewed. It should be noted that due to the methodological differences found across the studies in the review, and the relatively small number of studies identified (n = 13), a meta-analysis may have not yielded meaningful findings. Nonetheless, as more research on emotional eating and weight status in adolescents is published, a meta-analysis on this topic may be warranted. Another limitation of our systematic review was that studies were limited to those published or in-press in peer-reviewed journals in the English language. We also limited studies to those that utilized a self-report measure of emotional eating. It is likely that using different inclusion/exclusion criteria, including allowing studies that examined emotional eating in laboratory settings, would have yielded a larger number of studies which could have impacted our findings.

In conclusion, the results from our systematic review do not support an association between emotional eating and elevated weight status or reduced weight loss in adolescents. This systematic review highlights the need for more longitudinal research in this area with longer follow-up periods and more intervention studies that specifically evaluate weight loss in relation to emotional eating. The findings also demonstrate a need for more research examining the association between emotional eating and weight status among racial minority adolescents and the consideration of other constructs including loss of control eating.

## Figures and Tables

**Table 1:** Studies included in the systematic review.

Study	Emotional Eating Measure	Sample	Country of Study	Longitudinal	Association between Emotional Eating and Weight Status	Intervention
Hootman, K. C., Guertin, K. A., and Cassano, P. A. (2018).	Three-Factor Eating Questionnaire	241 college students 51.2% female Mean age = 18.1 years (SD = 0.3) Mean BMI for females = 21.5 (SD = 3.0) Mean BMI for males = 22.4 (SD = 3.1)	U.S.	Yes— mean follow up 14.1 weeks (SD = 1.1)	Longitudinal findings: No Baseline findings: Yes	No
Shriver, L. H., Dollar, J. M., Lawless, M., Calkins, S. D., Keane, S. P., Shanahan, L., and Wideman, L. (2019).	Three-Factor Eating Questionnaire	153 adolescents from the RIGHT Track study 56% female Multimethod longitudinal study at age 15 years, age 16 years, and age 19 years 64.1% European American, 29.9% African American, 3.6% biracial, 2.4% other race/ethnicity 68.8% in healthy BMI group, 15.6% in overweight BMI group, 15.6% in obese BMI group	U.S.	Yes—age 15, 16, 19 years	Yes	No
Simmons, S. and Limbers, C. A. (2019).	Emotional Eating Scale for Children and Adolescents	168 Latino middle and high school students 73.8% female Mean age = 13.69 years (SD = 0.88) Age range = 12–17 years 51.5% healthy BMI range, 23.3% overweight BMI range, 25.1% obese BMI range	U.S.	No	No	No
Snoek, H. M., Engels, R. C., van Strien, T., and Otten, R. (2013). Emotional, external and restrained eating behaviour and BMI trajectories in adolescence. *Appetite*, *67*, 81–87.	Dutch Eating Behavior Questionnaire	328 adolescents 51.5% female Mean age at wave 1 = 13.3 years Age range at wave 1 = 13–15 years 93% Dutch ethnicity	Netherlands	Yes—5 waves each one year apart	No	No
Stojek, M., Tanofsky-Kraff, M., Shomaker, L. B., Kelly, N. R., Thompson, K. A., Mehari, R. D., Marwitz, S. E., Demidowich, A. P., Galescu, O. A., and Brady, S. M. (2017).	Emotional Eating Scale for Children and Adolescents (EES-C)	156 adolescents 66% female Mean age at time 1 = 15.34 years (SD = 1.40) Age range = 13–17 years 61% White, 27% Black, 4% Asian, 8% Multiracial/Other Mean BMI = 24.33 (SD = 6.65)	U.S.	Yes—1 year follow up	No	No
Nguyen-Rodriguez, S. T., Chou, C. P., Unger, J. B., and Spruijt-Metz, D. (2008).	Dutch Eating Behavior Questionnaire	617 middle school students 75.8% female Mean age = 12.51 years (SD = 0.66) 61.7% Latino, 17.6% Asian, 17% Multiethnic or Other, 3.7% White 58% healthy BMI group, 42% overweight BMI group	U.S.	No	No	No
Järvholm, K., Olbers, T., Peltonen, M., Marcus, C., Dahlgren, J., Flodmark, C. E., Henfridsson, P., Gronowitz, E., and Karlsson, J. (2018).	Three-Factor Eating Questionnaire	82 adolescents from the Adolescent Morbid Obesity Surgery Study 67% girls Mean age = 16.9 years (SD = 1.15) Age range = 13–18 years Mean BMI at surgery = 45.4 (SD = 6.08) All participants had a BMI ≥ 35 at surgery	Sweden	Yes—2 year follow up after surgery	No	Yes
Mougharbel, F., Valois, D. D., Lamb, M., Buchholz, A., Obeid, N., Flament, M., and Goldfield, G. S. (2020)	Dutch Eating Behavior Questionnaire	1197 adolescents 60.3% female Mean age = 13.5 years (SD = 1.10) 70.3% healthy weight 18.1% overweight 5.1% obese	Canada	Yes—3 waves one year apart	No	No
Lowe, M. R., Annunziato, R. A., Markowitz, J. T., Didie, E., Bellace, D. L., Riddell, L., Maille, C., McKinney, S., and Stice, E. (2006).	Dutch Eating Behavior Questionnaire	72 college female students Mean age = 18.06 years (SD = 0.23) Age range = 18–19 years 76% Caucasian, 7% African American, 14% Asian American, 3% Hispanic American Mean BMI at time 1 = 21.9 (SD = 2.4) BMI range at time 1 = 17.4–26.6	U.S.	Yes—3 time points during freshmen year. The second and third data collection time points were approximately 4 and 8 months from the initial assessment	No	No
Laghi, F., Pompili, S., Baumgartner, E., and Baiocco, R. (2015).	Motivation for Eating Scale	336 adolescents 42% female Mean age = 17.48 years (SD = 0.50) Age range = 14–18 years Mean BMI for females = 21.33 (SD = 3.48) Mean BMI for males = 22.55 (SD = 3.31)	Italy	No	Yes, only for male adolescents	No
Snoek, H., Engels, R., Janssens, J., and van Strein, T. (2007)	Dutch Eating Behavior Questionnaire	428 adolescents 49.7% female Age range = 13–16 years 96% Dutch ethnicity	Netherlands	No	No	No
Gouveia et al. (2018)	Dutch Eating Behavior Questionnaire	572 adolescent–parent dyads 77.8% female Mean age = 14.34 years (SD = 1.59) Age range = 12–18 years 56.5% in healthy BMI group 43.5% in overweight/obese BMI group	Portugal	No	No	No
Zhu, H., Luo, X., Cai, T., Li, Z., and Liu, W. (2014)	Emotional Eating Scale	594 tenth- and eleventh-grade students 56.5% female Mean age = 16.7 years (SD = 1.09) Age range = 15–18 years BMI ranged from 16 to 29 Mean BMI for the sample = 20.06 (SD = 2.03)	China	No	No	No

## Data Availability

Not applicable.
